# A rare case report of dextrocardia combined with atrial fibrillation and ischemic bowel disease

**DOI:** 10.1097/MD.0000000000047189

**Published:** 2026-01-30

**Authors:** Yufei Zhao, Jiasong Li, Zhanjun Guo, Xiaoyun Zhang

**Affiliations:** a Department of Immunology and Rheumatology, The Fourth Hospital of Hebei Medical University, Shijiazhuang, Hebei, P.R. China.

**Keywords:** atrial fibrillation, dextrocardia, ischemic bowel disease

## Abstract

**Rationale:**

This report describes a rare case of dextrocardia concurrent with atrial fibrillation (AF) and ischemic bowel disease, to inform clinical management of such complex comorbidities.

**Patient concerns:**

A 70-year-old male presented with hematochezia, left lower abdominal pain, and AF confirmed by electrocardiogram.

**Diagnoses:**

Dextrocardia (complete transposition of great vessels), AF with atrial enlargement and left ventricular dysfunction, and colonic ischemic bowel disease (mucosal edema/ulceration) were confirmed.

**Interventions:**

Conservative therapy was given, including vasodilators, fluid resuscitation, anticoagulants, cardiac rate control, and antibiotics.

**Outcomes:**

Symptoms improved significantly posttreatment: abdominal pain resolved, stool color normalized, and cardiac status stabilized.

**Lessons:**

Comprehensive diagnosis and regular follow-up are essential for dextrocardia patients. Targeted intervention improves prognosis in complex comorbid cases.

## 1. Introduction

Dextrocardia is a rare congenital malformation where the heart is positioned in the right hemithorax, with both its base and apex oriented to the right.^[1,2]^ This intrinsic cardiac anomaly arises from embryonic development and is primarily classified into 2 types: situs solitus and situs inversus totalis.^[1]^ In situs solitus, only the heart is misplaced, while all other organs remain in their normal positions; this type is often associated with other congenital heart diseases (CHD) in 90% of cases. Conversely, situs inversus totalis involves both the heart and other organs being arranged as mirror images of their normal anatomical configuration; the anatomical relationship and structural integrity between the atria and ventricles are typically preserved; consequently, the anatomical relationship and structural integrity between the atria and ventricles are typically preserved, with only approximately 10% of patients developing CHD. The prognosis of dextrocardia is largely influenced by the presence of substantive pathological abnormalities or long-term sequelae.^[1]^

Atrial fibrillation (AF) is a common cardiac arrhythmia characterized by irregular and rapid atrial contractions.^[3]^ The complex pathophysiological mechanisms underlying AF include structural remodeling of the atria, inflammation, and dysfunction of the autonomic nervous system.^[4]^ A significant concern in AF patients is the heightened risk of blood clot formation, particularly within the left atrial appendage, due to stagnant blood flow caused by the loss of effective atrial contraction.^[5]^ These clots can detach and travel through the circulation, leading to embolic events.^[6]^

Ischemic bowel disease (IBD) is a condition marked by reduced blood flow to the intestinal wall, resulting in tissue damage.^[7,8]^ The descending colon, especially the splenic flexure, is particularly susceptible to ischemic insults due to its unique vascular anatomy and dependence on terminal arteries for blood supply.^[8]^ The main causes of mesenteric ischemia include embolic ischemia caused by AF and atherosclerotic narrowing of the mesenteric arteries, as well as nonocclusive ischemia caused by impaired cardiac output and peripheral perfusion.^[7]^ Consequently, IBD is prevalent among older adults with underlying cardiovascular diseases, with over 90% of cases occurring in this demographic.^[9]^

The concurrent occurrence of dextrocardia, AF, and IBD presents significant challenges for clinical diagnosis and management. This article reports a rare case of a patient with these 3 conditions simultaneously, discussing the clinical features, treatment approach, and prognosis associated with this unique presentation.

## 2. Case report

A 70-year-old male patient, who has a congenital condition of dextrocardia, was admitted to Hebei Medical University Fourth Hospital in February 2024 due to bloody stool. 15 days prior to admission, the patient experienced episodes of watery melena following exposure to cold weather, with an average of 3 episodes daily, each discharging approximately 50 to 100 mL of melena. Subsequently, the stools became bloody with intermittent left lower abdominal spasmodic pain. There were no other associated symptoms such as fever, vomiting blood, dizziness, syncope, or palpitations. He had a 20-year history of hypertension, which was controlled through regular medication, and denied any histories of diabetes, coronary heart disease, hepatitis, tuberculosis, or other significant illnesses.

**Figure 1.**
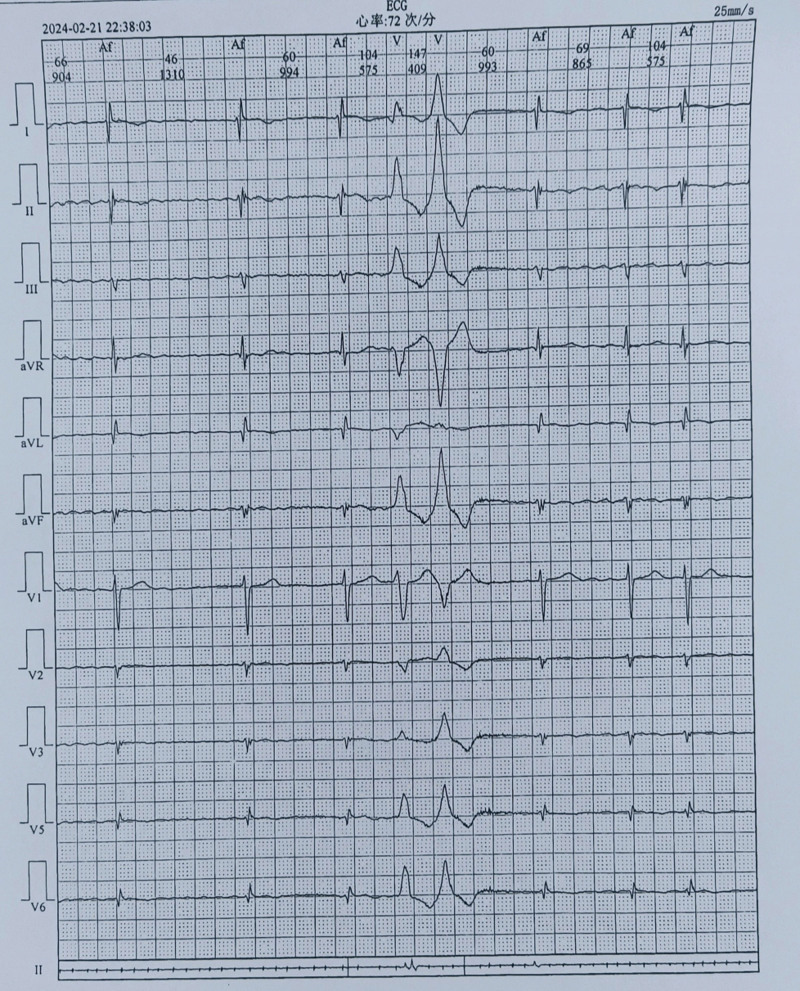
Twelve-lead 24-hour ambulatory electrocardiogram.

**Figure 2.**
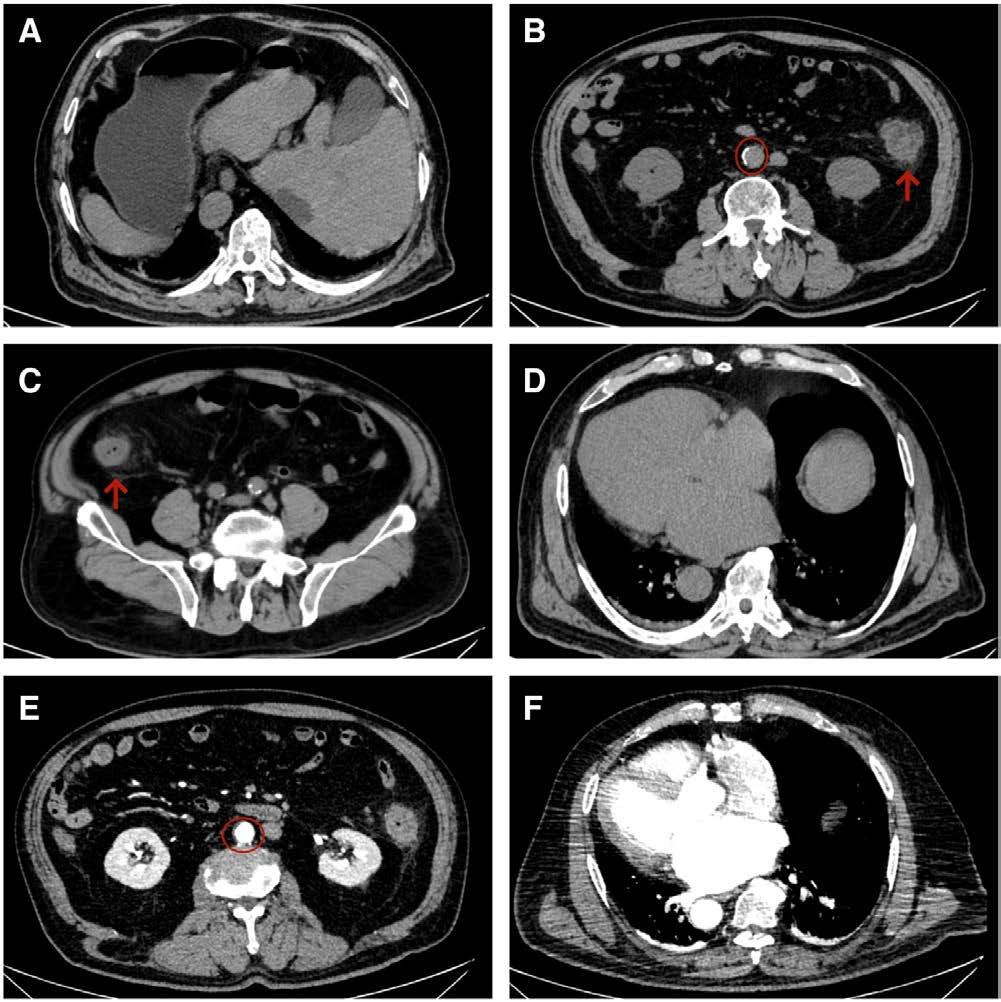
CT and CTA images. (A) CT scan results showed situs inversus totalis, localized thickening of the (B) left and (C) right colonic walls with blurred surrounding fatty spaces (red arrow), (D) and arc-shaped increased density shadows beneath the pleural membranes of both lower lung lobes. (E) Abdominal CTA revealed arteriosclerotic changes (red circle), (F) no abnormalities were detected in pulmonary CTA. CT = computed tomography, CTA = computed tomography angiography.

**Figure 3.**
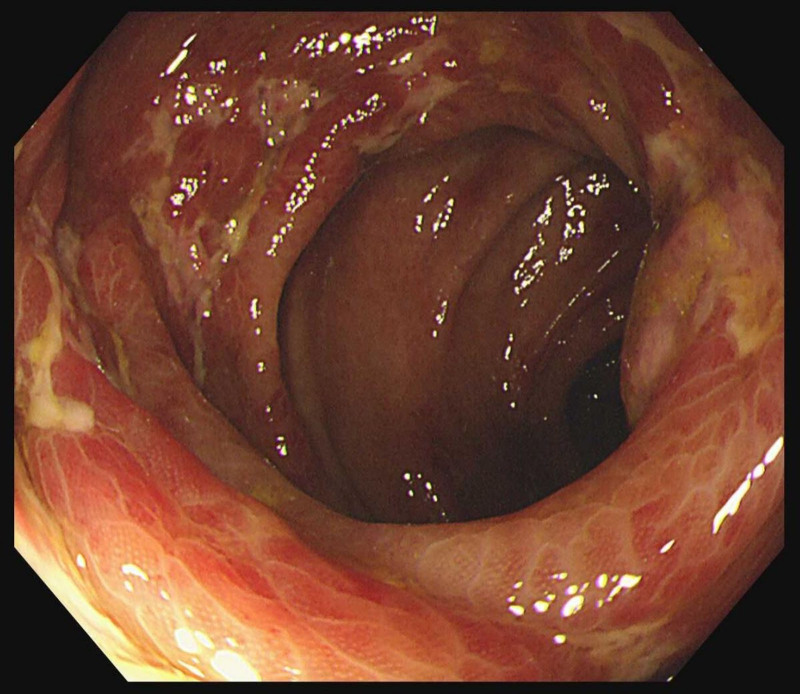
The colonoscopy shows diffuse hyperemia, erosion, and superficial ulcers in the descending colon near the splenic flexure.

Upon admission, vital signs were stable with a temperature of 36.4 °C, respiratory rate of 18 breaths per minute, and blood pressure of 115/77 mm Hg. The abdominal examinations showed a soft abdomen without tenderness, and bowel sounds were active; however, the cardiac auscultation revealed completely irregular heart rhythm, varying heart rates, unequal heart sounds, and a pulse rate of 66 beats per minute, which was slower than the heart rate of 85 beats per minute. The 24-hour, 12-lead ambulatory electrocardiogram demonstrated AF (totaling 103,404 heartbeats with an average ventricular rate of 74 beats per minute), prolonged R-R intervals (the longest measuring 2.5 seconds), and occasional premature ventricular contractions (comprising 783 single beats and 4 paired beats) (Fig. 1). Laboratory tests showed a N terminal pro-brain natriuretic peptide level of 3510.00 ng/mL (normal range, 0–299 ng/mL), a white blood cell count of 19.61 × 10^9^/L (normal range, 3.5–9.5 × 10^9^/L), a hemoglobin level of 167 g/L (normal range, 130–175 g/L), a platelet count of 189 × 10^9^/L (normal range, 125–350 × 10^9^/L), a D-dimer level of 1.174 mg/L (normal range, <0.243 mg/L), and a procalcitonin level of 0.14 ng/mL (normal range, <0.05 ng/mL). No significant elevation in tumor markers associated with colorectal cancer was observed. Ultrasonic cardiogram revealed dextrocardia, enlargement of the left atrium with mild mitral regurgitation, degenerative changes in the aortic valve with mild insufficiency, impaired left ventricular diastolic function, atrial fibrillation, and a left ventricular ejection fraction of 54%. Computed tomography scan results showed situs inversus totalis, localized thickening of the left and right colonic walls with blurred surrounding fatty spaces, and arc-shaped increased density shadows beneath the pleural membranes of both lower lung lobes. Additionally, abdominal computed tomography angiography (CTA) revealed arteriosclerotic changes, while no abnormalities were detected in pulmonary CTA (Fig. 2). During the colonoscopy, scattered congestion, erosion, and superficial ulceration were observed in the colonic mucosa, with the most significant involvement in the descending colon near the splenic flexure, presenting a diffuse pattern (Fig. 3). The pathological examination of the descending colon via sigmoidoscopy revealed chronic mucosal inflammation. Ultimately, the patient was diagnosed with situs inversus totalis, atrial fibrillation,^[3]^ and IBD.^[10]^

In the subsequent management, a nonsurgical treatment approach was implemented as an integral part of the overall strategy for IBD. The patient was initially placed on nil by mouth status and underwent fluid resuscitation to ensure hemodynamic stability. Following this, the patient received an intravenous injection of 2 units of thromboplastin as an initial intervention. Subsequently, a regimen of 30 mg papaverine was administered via intramuscular injection every 8 hours, coupled with intravenous infusions of a third-generation cephalosporin antibiotic every 12 hours. By the third day following treatment, the patient experienced a notable reduction in abdominal pain, and the stool returned to a normal yellow color. Then, to further mitigate the risk of thromboembolic events, the patient was prescribed low-molecular-weight heparin at a dose of 6000 units via subcutaneous injection every 12 hours. Addressing the patient’s AF and heart failure, a combination therapy was initiated, comprising a β-blocker, a neprilysin inhibitor, an angiotensin receptor blocker, and a sodium-glucose cotransporter-2 inhibitor.

On the 5th day following treatment, the patient was discharged from the hospital. At discharge, laboratory results indicated a N terminal pro-brain natriuretic peptide level of 1350.00 ng/mL (normal range, 0–299 ng/mL), a white blood cell count of 5.24 × 10^9^/L (normal range, 3.5–9.5 × 10^9^/L), a D-dimer level of 0.198 mg/L (normal range, <0.243 mg/L), and a procalcitonin level of 0.05 ng/mL (normal range, <0.05 ng/mL). However, the electrocardiogram continued to reveal persistent AF. As of the writing of this case report, the patient has been consistently adhering to an oral Factor Xa inhibitor regimen for anticoagulation therapy. There have been no recurrences of abdominal pain, hematochezia, or palpitations; however, a follow-up colonoscopy has yet to be conducted.

## 3. Literature review

### 3.1. Research progress on dextrocardia

Dextrocardia is a relatively rare congenital cardiac malformation, with an estimated incidence of approximately 0.83 per 10,000 pregnancies.^[11]^ Based on cardiac anatomy and the positional relationship of thoracoabdominal organs, it is primarily classified into 2 types: mirror-image dextrocardia (situs inversus totalis) and isolated dextrocardia (situs solitus).^[1]^ In mirror-image dextrocardia, the heart is located in the right hemithorax, and the positions of thoracoabdominal organs such as the liver and stomach are completely reversed, presenting as a mirror image of the normal anatomy. The connections of the cardiac atria, ventricles, and great vessels are usually normal in these patients, and the probability of associated CHD is relatively low, around 10%. In contrast, patients with isolated dextrocardia (situs solitus) have only the heart located in the right hemithorax with normal positioning of other organs; however, about 90% of these cases are associated with other congenital cardiovascular malformations, such as transposition of the great arteries, pulmonary stenosis, and atrial or ventricular septal defects. Relevant studies suggest that the pathogenesis of dextrocardia may be related to abnormal rotation and displacement of the cardiac loop during embryonic development.^[12]^ In recent years, imaging techniques have played a key role in the diagnosis of dextrocardia. Echocardiography with contrast can clearly display the internal cardiac structure and hemodynamic changes, while CTA can intuitively reveal the spatial positional relationships of the heart and great vessels, providing crucial basis for clinical diagnosis and treatment planning.

### 3.2. Research on the association between AF and IBD

AF is a common clinical arrhythmia with a complex pathophysiological mechanism involving atrial structural remodeling, inflammatory responses, and autonomic nervous system dysfunction. During AF, the effective contractile function of the atria is lost, leading to blood stasis within the atria, particularly in the left atrial appendage, which predisposes to thrombus formation.^[13]^ Statistics show that the risk of thromboembolism in AF patients is significantly increased compared to the normal population. Due to the anatomical characteristics of the intestinal vasculature, such as the long vascular arcade of the superior mesenteric artery with numerous branches and relatively slow blood flow, thrombi associated with AF can easily embolize the mesenteric vessels, causing IBD.^[14]^ Clinical studies have found an increasing incidence of IBD among AF patients, particularly evident in the elderly population and those with other comorbid cardiovascular conditions.^[15]^ In-depth research into the association between AF and IBD is crucial for the early identification of high-risk patients, enabling timely interventions such as anticoagulation therapy to reduce the risk of IBD occurrence.

### 3.3. Case reports on the co-occurrence of dextrocardia, AF, and IBD

No combined cases of dextrocardia, atrial fibrillation, and IBD have been reported to date. Due to the complexity of the condition, management requires multidisciplinary collaboration involving cardiology, gastroenterology, radiology, and other specialties. Diagnosis primarily relies on a combination of various examinations, including cardiac ultrasound, CTA, and colonoscopy. Treatment must comprehensively address multiple factors, such as anticoagulation for atrial fibrillation, improvement of intestinal blood supply, and infection control. However, owing to the limited sample size (i.e., the rarity of this combined condition), there is currently insufficient clinical evidence regarding the pathogenesis, optimal diagnostic and therapeutic strategies, and prognosis evaluation for this combined condition. Further case accumulation and in-depth research are urgently needed.

## 4. Discussion

This report presents a case involving a patient with concurrent dextrocardia, AF, and IBD. To the best of our knowledge, there are currently no documented cases of coexistence among these 3 diseases, and we have proposed potential mechanisms that may underlie this. The alteration in heart position resulting from dextrocardia may impact cardiac hemodynamics, leading to an uneven distribution of cardiac load. Over time, this can elevate the risk of structural and functional abnormalities in the heart, potentially leading to the onset of AF. In the state of AF, the effective contractile function of the atria is lost, causing blood to stagnate within the atria, particularly in the left atrial appendage, a region with complex structure and slower blood flow, where the atria are highly susceptible to vortex formation and subsequent thrombus development.^[5]^ Once these thrombi detach, they can travel through the bloodstream and enter the intestinal blood supply vessels, particularly those in the descending colon where the vascular network is relatively intricate, dense, tortuous, and branched, making them prone to mesenteric embolism.^[16]^ Following mesenteric embolism, the blood supply to the intestine is drastically reduced, leading to tissue hypoxia and necrosis, thereby triggering IBD.^[7,17]^ Additionally, concurrent cardiac dysfunction in these patients impairs the heart’s pumping ability, further compromising intestinal blood perfusion and exacerbating the ischemic state of the gut. Moreover, the presence of atherosclerotic plaques in these patients poses a risk of rupture, releasing platelets and other coagulation factors, which further promote thrombus formation and embolism.

The correlation between dextrocardia and cardiac arrhythmia remains controversial. Some scholars argue that since situs inversus totalis represents a complete mirror-image arrangement of the visceral organs relative to their normal positions, the connections between the atria and ventricles, as well as the heart structure, are theoretically normal.^[1,2,18]^ However, previous retrospective studies and case reports documented occurrences of atrial flutter and sick sinus syndrome in patients with situs inversus totalis, suggesting that dextrocardia may harbor potential defects in the cardiac conduction system.^[2,9,19]^ Further empirical evidence is required to substantiate this relationship.

In the current case report, the exact onset of AF remained unclear, and the patient presented with no discernible clinical symptoms. Consequently, neither pharmacological nor radiofrequency ablation was attempted for electrical cardioversion; instead, β-blocker and anticoagulation therapy were chosen as the primary interventions to control ventricular rate and prevent thromboembolic events. Concurrently, we prompted a comprehensive treatment strategy for IBD that encompassed vasodilators, anticoagulation, antibiotics, fluid restriction, and fluid resuscitation. The patient responded favorably to this treatment strategy, with a notable improvement in symptoms within 3 days following treatment. By documenting this successful treatment outcome, we aspire to contribute valuable insights into the diagnosis, management, and prognosis of such complex cases.

This study has the following inherent limitations, and its clinical reference value should be viewed objectively: first, this report only includes one patient, and the patient has unique clinical characteristics which cannot represent the overall patient group with such combined diseases. Second, the depth of mechanism exploration is limited. Although the patient’s diagnosis and treatment process was recorded in detail, due to the case study design, it was not possible to conduct a control analysis or molecular biological testing. Only clinical phenomena could be used to infer the association among the 3, making it difficult to verify the causal relationship between dextrocardia and atrial fibrillation and intestinal ischemia and to clarify the specific causes of thrombosis and the pathological and physiological details of intestinal ischemia in this case. Third, the follow-up period and data are incomplete. This report only presents the short-term diagnosis and treatment outcomes of the patient, lacking long-term follow-up data.

## 5. Conclusion

This case report provides evidence supporting a potential link between dextrocardia and AF. Notably, it marks the first documentation of the concurrent presentation of AF and IBD within the rare patient population with dextrocardia (an observation that adds new insights to the clinical understanding of comorbidities associated with this anatomical anomaly). For patients with dextrocardia, regular follow-up and adherence to treatment plans are crucial to minimize the risk of complications.

## Author contributions

**Conceptualization:** Xiaoyun Zhang.

**Data curation:** Jiasong Li.

**Investigation:** Jiasong Li.

**Project administration:** Zhanjun Guo.

**Resources:** Zhanjun Guo.

**Supervision:** Zhanjun Guo.

**Writing – original draft:** Yufei Zhao.

**Writing – review & editing:** Xiaoyun Zhang.
